# Nurses' Experiences of Caring for Older Persons in Transition to Receive Homecare: Being Somewhere in between Competing Values

**DOI:** 10.1155/2013/181670

**Published:** 2013-05-16

**Authors:** Sigrun Hvalvik, Bjørg Dale

**Affiliations:** ^1^Faculty of Health and Social Studies, Telemark University College, 3901 Porsgrunn, Norway; ^2^Centre for Caring Research-Southern Norway, Faculty of Health and Sport Sciences, University of Agder, 4898 Grimstad, Norway; ^3^Centre for Caring Research-Southern Norway, Department of Health Studies, Telemark University College, 3901 Porsgrunn, Norway; ^4^Faculty of Health and Sport Sciences, University of Agder, 4898 Grimstad, Norway

## Abstract

Older persons in transition to need professional care in their homes will constitute a large group in municipalities in the future. The aim of this study was to obtain insight into nurses' experiences and perceptions of caring for patients in transition to receive homecare. Eleven home nurses divided into two focus groups were interviewed, and a phenomenological hermeneutical design was used. Four interpretations closely related to each other were revealed: it is essential to have an understanding of the patients' transition history; the nurse' repertoire is challenged in the transition process; care must be adapted to the patients' life world; the excellence of care is threatened by the context. The nurses strived to provide care based upon respect for the independent individual as a living whole. Their ambitions were, however, challenged and threatened by the caring context. The cooperation across organizational levels was pointed out as a critical factor with potential for improvement. This must be taken seriously to support the nurses in their endeavors to provide excellent care.

## 1. Introduction

The transition from independence to needing professional help to manage daily self-care may bring about various types of distress and vulnerability to an individual. To receive the professional support needed in such situations, a healthy transition is vital [1]. Older persons in transition to need professional care in their homes will constitute a large patient group in Norwegian municipalities in the future, and many of them will have increasing needs for coordinated services [2]. In this respect, home care services and nurses working close to the patients in practice are crucial. They are key persons for enabling healthy transitions for older persons in need of home care and for dealing with challenges related to meeting the care needs of this patient group.

As in other Western European countries, the demographic and epidemiological patterns in Norway are undergoing great changes. The number of older people expands, and, simultaneously, the number of persons with chronic and complex illnesses in this group expands [2]. One consequence of this development is that the amount of older persons in need of professional care in their own homes is increasing. While approximately 40,000 persons live in nursing homes today in Norway, more than 160,000 individuals receive municipal home care services, either in their private residences or in assisted living facilities [3]. This number is supposed to further increase after the Coordination Reform was brought into force in January 2012; as a major focus in this reform is that patients shall receive proper treatment at the right place and at the right time [2]. One goal in this respect is that older patients shall be quickly discharged from the hospital and return to his or her residential municipality after completing treatment in the specialist care. To provide good quality care to this group of patients in transition, as well as to older persons in other types of transition to receive professional care in their homes, is challenging and highly competent health care providers are required.

Many studies show that living in their own homes is preferred by the elderly themselves [4–6]. Further, older persons' self-care ability and health are shown to be significant factors for managing daily life in their own homes and being satisfied with their life [7, 8]. Professional care that contributes to promoting both self-care and health status is therefore of utmost importance, first and foremost for the individuals themselves, and for society as well. These aims are also in accordance with health care policy concerning older people, on a national as well as an international level [2, 3, 9].

Nordenfelt [10] claims that when a person is no longer able to act or care for himself or herself independently, it may result in lower sense of self-worth. To be dependent upon professional care in the daily life may therefore be a critical point in life, and it may make a person in transition to receive homecare vulnerable. This is in accordance with the findings in a previous study where older home dwelling people in transition from self-supported to supported living were interviewed about their perceptions of the actual transition process [11]. According to Meleis and Trangenstein [12], one of the goals in nursing from a transition perspective is to facilitate the transition process so that health and wellbeing is the outcome. To assist people in the transition towards a sense of mastery as illness and changes disrupt their lives is consequently vital in nursing [13]. Therefore, to obtain insight into nurses' experiences and perceived care for older persons who are in transition to receive professional help in their homes is of great importance. This insight will also contribute to a deeper understanding of the concept of transition from independence to needing professional care.

The authors have not been able to identify former studies focused on nurses' experiences and perceptions from the particular perspectives described above. Studies performed between 2000 and 2012 concerning nurses' perceptions of care for older people have primarily focused on care and the quality improvement of care, in institutional long-term settings [14–17]. In addition, nursing studies concerning older persons' transitions in the same time period have mostly focused on various types of relocation, either between different levels of care, from private residences to nursing homes, or to assisted-living facilities [18–21]. There are also several studies concerning transitional care which are mainly focusing on the coordination and the continuity of health care services to patients transferring between different locations or different levels of care in the same location [22–24]. Although these studies are of interest and are relevant for the present study, they have emphasized other aspects. 

*Aim. *The present study examined the everyday life world of nurses working in a home care setting focusing on the care for older persons in transition to receive professional health care in their homes. The aim of the study was to define nurses' experiences and perceptions of providing care to older persons in transition to receive professional care in their homes.

## 2. Materials and Methods

### 2.1. Design

To explore the nurses' experiences of caring for older persons in transition to receive homecare, a phenomenological hermeneutic design was chosen. In both phenomenology and hermeneutic philosophy, the life world perspective, focusing on how the world with its everyday phenomena is lived, experienced, acted, and described by humans, is fundamental [25]. Leaning on this perspective means we believe that individuals and their existence can never be satisfactorily understood if they are not looked upon as living wholes. Consequently, the picture given by the participants in this study of those who provide the patients professional care in their homes would not be complete if their own experiences and self-attendance were not taken into account [25]. The hermeneutical approach is influenced by the German philosopher Gadamer [26], based upon his assumptions of preunderstanding, the hermeneutic circle and openness. According to Gadamer, the researcher has to identify his own preunderstandings of the phenomenon under investigation, which means being conscious of and reflecting upon different scientific and theoretical presuppositions and personal views [26]. This consciousness will enable the researcher to go beyond her or his own preunderstandings to understand the subject and in this way exceed her or his own horizon. In this sense, consciousness provides openness to see and understand the phenomenon in new ways. Openness is critical in waiting for the phenomenon to reveal its complexity and to leave behind any of the expectations for what to be found [25]. Therefore, this approach makes it vital to handle knowledge and preunderstandings of the subject under investigation in ways that allow a new understanding to emerge. This attitude has influenced the entire research process in our study. 

### 2.2. Study Group

To achieve a deeper understanding of how nurses experienced and perceived the care of older persons in transition to receive professional care in their homes, a purposive sampling method was used to recruit participants for focus group interviews. The home care offices in two municipalities in southern Norway were informed about the project, and registered nurses who had been working in home care services for at least one year and more than 50% employed were subsequently invited to participate in the study. The total sample included 11 participants, which were geographically divided into two focus groups, one consisting of five members, the other consisting of six members. All participants were working closely with the patients.

### 2.3. Data Collection

Focus group interviews were preferred as the data collection method in this study. Focus group interviews are relevant when phenomena involving common experiences, attitudes, or views in a context characterized by cooperation between numbers of people are investigated [27]. The purpose of the interviews was to obtain in depth information about the nurses' experiences and perceptions about caring for the actual patient group. Thus, we considered focus group interviews to be convenient for this purpose as the participants are allowed to share experiences and views of the phenomenon. Each group met once. The two researchers were present in both group interviews, changing the roles as moderator and secretary.

The initial question in both interviews was what kind of experiences and perceptions do you have regarding care for patients who are in transition to receive professional care in their homes? The participants were encouraged to describe positive as well as negative experiences and to expand or explain their views. Both researchers were also experienced nurses and had a basic understanding of the phenomenon under investigation. This was important for asking relevant follow-up questions through the interview process and also to continuously keep to the phenomenon under investigation in focus. The researchers had previously discussed and reflected upon the subject for the interviews, which provided them with possibilities to move beyond their preunderstandings and to transcend their understandings during the focus group interviews [26]. In this way, their attitudes were characterized by openness and flexibility in the interview situation. 

The focus group interviews lasted approximately 1–1.5 hours. They were audio taped and transcribed verbatim.

### 2.4. Data Analysis

Initially in the analysis process, both researchers individually listened to the tape and read the interviews as whole texts in order to obtain a first-hand understanding of the fundamental meaning of the text as a whole. Before they met for further discussions, they had made a preliminary structure according to themes in the interviews. Together they went carefully through the texts once more for further reflections and identifications of themes and variations over the themes [25, 28]. The researchers moved critically back and forth between the themes and the variations in order to develop interpretations on a higher and more abstract level. Finally, the interpretations of the textual content were compared in order to identify a primary interpretation reflecting significant aspects regarding the care of older persons in transition to receive home care. This primary interpretation was related to the identified interpretations in various ways [25]. 

### 2.5. Ethics

The study was approved by the Norwegian Social Science Data Services on 17 April 2012. All participants were given both oral and written information. Confidentiality and voluntariness for participating in the study were assured, as well as the participants' right to withdraw from the study at any time if they wanted. Written informed consents were obtained before the study began. 

## 3. Results

The transcribed focus group interviews were analysed as a whole. Four distinct interpretations, closely related to each other, were identified: it is essential to have an understanding of the patients' transition history, the nurse' repertoire is challenged in the transition process, care must be adapted to the patients' life world, and, the quality of care is challenged by the context. These interpretations, as represented in Table 1, were not only based upon narratives of practice experiences but also on the participants' general perceptions and views of care.

### 3.1. It Is Essential to Have Insight into the Patients' Transition History

To have an insight into the patients' transition history meant to have comprehensive knowledge about why the older person had to start receiving homecare and to have insight into the history related to this event. The participants described this to be fundamental, and they underlined the fact that patients in transition to receive care in their homes had very different stories connected to the reasons for needing homecare. The participants paid a lot of attention to these stories. First, they described how the causative factors, or the marker events that caused the need for daily professional support, affected the patients' lives in different ways. The patients' individual situations and initial positions varied, and the nurses emphasized the importance of obtaining insight into these circumstances. At the moment when the older persons began homecare many of them already had been in a transition process for longer or shorter time, and this process had often been both complicated and complex. Some of them had been in need of help for a long time but had hesitated to contact the health care services. One of the participants reflected “I think the process to realize that you need help is hard and painful and that the steps before contacting the homecare often are too many.” Other patients had been acutely ill and hospitalized or their chronic illnesses had deteriorated and made hospitalization necessary, and after the hospital discharge they needed further help and support in their homes. These patients were often confused and lacked information about their hospital stay and some of them had not been involved in the transition process regarding homecare at all. One category of patients who often represented particularly challenging transition situations included those who had, more or less, been forced by their next of kin to start receiving home care against their own preferences. Thus, these patients often felt run over by their relatives in a way that threatened their autonomy and independence. Another category of patients who was mentioned as particularly exposed to problematic transitions was those who were in an initial phase of cognitive impairment. These patients were vulnerable because they often forgot that they had started receiving home care or for what reasons, and often they did not recognize the nurses when they came. Consequently, the whole situation made them unsecure and confused. One of the nurses told a story about a woman who, in spite of a beginning cognitive impairment, had managed on her own. However, when she broke her wrist, she became in need of daily help for a period. She had lived her life in total solitude for years and was afraid of people, and, hence, she would not let the nurses in and claimed that she could manage herself. However, from the nurses' view, she definitely needed help to perform her daily life activities. Consequently, this transition to needing home care became problematic and challenging for all parties. 

However, while some of the older persons preferred to avoid help from the home nurses, others were happy to receive it. Accordingly, the patients' health status and need for help as well as their need for information varied, and so did their attitudes towards receiving help. To get a comprehensive insight into these types of vital conditions, the nurses had to provide knowledge from all available sources, as well as listen to the experiences from both the patients and the next of kin. One of the participants explained “To get that background information, what is the diagnosis, what is the problem…to be familiar with the patient, I think it's fundamental for further care.” 

Information about the patients and their transition history made it possible to see the present situation in a larger context. This was decisive to achieve a preliminary understanding of how their lives had been changed and how and why they perceived their situation as they did. To obtain this kind of understanding was regarded as significant in identifying and understanding their specific needs, as well as having empathy with and comfort for them in their situation. To meet the patients in this initial phase in a competent way generated trust and was crucial to establish a relationship with the patient. A trusting relationship was described as the very fundamented for further care and for gaining a healthy transition process: “The more the person in concern trusts you, the more information you get and the better help you are able to give.” 

### 3.2. The Nurse' Repertoire Is Challenged in the Transition Process

The participants defined themselves and their caring actions as intertwining between their qualities as human beings and as professional nurses. These qualities constituted a type of repertoire that determined their caring actions as well as the way they performed care. In the initial phase of providing home care to older persons the nurses' repertoire was challenged in several ways. The participants had experienced that patients in this phase of the transition process were especially sensitive to the nurses' attitudes and that the patients had a strong wish to please them. If, for instance, the nurse more or less unconsciously gave the impression of being busy, the patients hesitated to use the time they needed to perform the self-care tasks by themselves and thereby to regain these such as dressing and buttoning up. The interviewees described that the nurses, in this way, could signal that the patient was a burden that “took the nurses' precious time”. One of the informants stated “I'm a kind of restless person, when I do the things for the patient, it goes so much faster. It's not that I haven't got the time. But it is me as a person.” To be aware that your own personality was reflected in and could affect the performance of care in a negative as well as a positive way was therefore crucial.

The participants described how providing care in the patients' own homes gave them valuable opportunities to be more personal and familiar with the patients: “…you know, we have some golden opportunities to talk with them as fellow beings in a way, for example, about pictures hanging on the walls in their homes, you may come in and…and confirm that they are still the same persons they were before.” “To see the person in the patient,” as they expressed it, made it easier to comfort them as well as to confirm their identity. To be aware of and take advantage of these types of opportunities was particularly important in the transition process. When they acted as fellow-beings the participants also felt that they met the patients' psychosocial needs in a natural and essential way. Having the patients' homes as their working place made it possible to extend the nurses' repertoire of actions, which they described as vital to take advantage of in the transition process. 

Another perspective in the interviews was the significance of having professional courage to engage with and also strategies to handle situations related to the next of kin. Although they represented an important resource, the participants had a range of experiences with next of kin in the actual transition process which could generate various types of dilemmas. Next of kin could have opinions and experiences that differed from those of the patients', and sometimes they also “run over” them in unacceptable ways. In these cases, the nurses had to stand up for the patients and argue as professionals on their behalf. To be professional in situations like this, however, also meant to explore and consider the situation and to have the courage to discuss and find “a golden middle way” and an acceptable solution. This could be especially challenging when the patient suffered from cognitive decline: “We have to communicate in such ways that no one gets hurt, but rather understand us as professionals. I think that is, very important, especially in the initial phase. …I think we have to take that conversation with the relatives to find a purposeful solution for the person in need of help as well as for the next of kin. That's why we are there.” To find good strategies in situations like this was considered most valuable, as they also considered the cooperation with next of kin of utmost importance. For the same reason, the nurses had to make clear to the next of kin what they could expect of them as health care providers and on which basis and to what extent the nurses could make decisions in caring situations with the patient.

 Another aspect that the participants reflected upon was the nurses' ability and preparedness to act and make decisions when necessary in the context with older persons in transition. Idealistically, the nurses should be prepared to meet the patients in transition and act in a planned and purposeful way. In this sense, they had to acquire sufficient knowledge about the patients and their total situation and adequate skills to arrange for patient care of high quality. However, sometimes this was impossible if they, for some reason or another, lacked information about the patient. In such cases, they still had to demonstrate competence and self-governance to deal with the situation and act to meet the patients' needs. To manage this meant to have knowledge and capability to make independent priorities and choices, to be attentive to the patients' needs, and to act in correspondence with the actual situation. 

An overall ambition in the care for patients in transition was to behave and act in such a way that the patients experienced as noninvading: “To have the time to…, to be there in a way, yeah, to meet the patient “at home” in a way…To be and to act in a way that put you in a position to really help. It can be very hard, but oh so great when you succeed!”. 

### 3.3. Care Must Be Adapted to the Patients' Life World

The importance of meeting the basic needs of the patients in transition in an individual and person oriented way was another key perspective that was emphasized by the interviewees. This aspect was enlightened through the participants narratives as well as through their described ideals of caring.

 In the participants' view, excellent care had to be based on the patients' habits in their everyday life. To succeed in this sense, time and continuity in the relationships with the patients and next of kin were vital aspects. This gave them opportunity to achieve insight into how the patients' lives had been organized and lived prior to being in need of professional help in their homes. In this way, the nurses endeavoured to regard the patients' care needs, as well as their experiences of the changed situation, in the context of their life world: “To care for each individual, to understand how he has been in a way…to see the whole patient.” In the extension of this, the participants expressed that they had to gain knowledge about how the patients used to perform their own self-care. This was necessary for adapting the professional care to the patients' habits: “We try to organize and be as flexible as possible in a way—let them continue in their habits—in a way … try to have respect for their time as well in a way.” As far as possible they supported the patients in their own ways of practicing, even if it might be difficult. However, if, for instance, the patients' hygiene habits were unfavourable with respect to their diagnosis and state of health, the nurses tried to convince the patients through information that they ought to change their practices. Sometimes situations like these could constitute challenging dilemmas often associated with the patients' autonomy. The participants expressed that if such dilemmas arose, they made great efforts to come to terms that both parties could accept.

The patients' resources were another aspect that was highly emphasized by the informants, and they did not regard the patients' resources solely related to the patients' state of health and care needs. Rather, another important perspective related to the patients' resources was their long lived lives as human beings: “What can they perform themselves, what do they want to manage themselves and to be sure that the home care services, or that I, as a nurse, do not go in there and step on their life experiences, step on their knowledge, step on what they have of resources. It is so important!”. In this way, the participants regarded the patients' resources in a broader perspective which included those gained through a long life course. Consequently, resources to manage self-care were considered only as a part of the patients' total resources. This in fact made it impossible to compare the patients' vulnerability measured through resources or lack of resources to manage self-care. One of the participants described “Those who have resources to manage themselves may be just as vulnerable as those who do not manage. It all depends on the person. And the vulnerability of those with resources might be even more difficult to discover.” The participants, however, agreed that it was of great importance to stimulate and utilize the patients' self-care resources, and they also described how they used the patients' homely context to motivate the promotion of self-care. This meant, for example, to follow the patient to the post box to collect the mail, instead of collecting the mail for her or him. 

Working in the patients' home often meant to relate to next of kin. The participants described this relationship as more or less complex depending upon the interaction between the patient and next of kin. Basically, they had to take the next of kin into account when caring for the patient. In addition, often the next of kin had their own needs which the nurses had to attend to, such as support, information, and comfort. Sometimes the patients were totally dependent upon their next of kin to continue living at home. The more present the next of kin were in the patients' lives, the more significant they became in the care provision. Thus, care that was designed to meet the fundamental needs of patients in transition in an individual and person oriented way had to take “the life of the whole patient” into account. This involved their everyday life as well as their former life experiences.

### 3.4. The Quality of Care Is Challenged by the Context

The participants described planning and performance of care that was person oriented and directed towards the promotion of self-care and wellbeing as a particularly important goal related to patients in transition to receive home care. However, they experienced that several contextual conditions challenged and threatened the aim and quality of care, such as lack of coordination, competence, information, continuity, and time. They perceived that patients in transition were particularly vulnerable to these challenges.

The cooperation between the several levels and agencies in the health care system was mentioned as decisive regarding patients in transition. They claimed that lack of communication and insight into each other's tasks and responsibilities on different levels and areas influenced the cooperation in a negative way. A subject of great concern was that the health services were arranged in such a way that the home care professionals had to provide care tasks to the patients in transition which were composed of and defined on another level in the system. While some of the participants experienced this as mainly unproblematic, more of them found it problematic. They explained that care composed beforehand by other persons could be deficient, have an irrelevant focus, be founded upon secondary sources, or be based upon available resources in the system rather than on the needs of the patients. The care could also be unrealistic to accomplish, or be based upon information in conflict with other types of medical reports. And, in the worst case, the information could be missing. These circumstances were described to influence the quality of care in negative ways and revealed that the communication between the levels failed. One of the participants described it this way: “I think that the communication between the various levels is very diffuse, because very often…it is, strictly speaking, they who compose and define the care and are responsible for the very first assessment and registration, but very often they haven't done it, and then we have to take it ourselves.” 

The cooperation between the home care services and the specialist health care was described by all of the participants as challenging. The greatest challenge was related to the routine of discharging the patients, who were in transition to receive homecare, on Friday afternoons, which happened to be quite usual. The patients often returned to their own home without necessary medicines, and often they also lacked prescriptions or the helping aids they might need. As one of the participants expressed: “Well, then the system is down, and if you need more medicines, what do you do then? The primary doctor's office may be closed, and does not open until Monday. …Well, this could be a really bad start for both the patient and their relationship with the home care services.” Additionally, the home care services often were badly staffed on the weekends. There were fewer persons on duty and were often those with the lowest competence. This meant, among other things, that the nurses lacked the necessary time to talk with the patient and to get important information. The participants described this situation as really unfortunate as this group of patients in transition often were confused about their condition and diagnosis. They needed information and to talk about what they had been through as well. It also varied what type of information they received and what types of expectations they had for the home care services. To deal with all these challenges, the nurses had to spend time and talk with the patients. Therefore, the discharge of patients in need of homecare on Friday afternoons was most unfortunate for all parties. As one of the participants expressed: “Well, then you are, as you say, on thin ice as regards the relationship of trust.” 

All the participants stressed the significance of getting familiar with the patients and identifying their needs in the initial phase of the transition process. They also emphasized the continuity by means of having as few nurses as possible to care for the patient in the transition process. Three conditions were mentioned as hindrances in this sense: time, continuity, and competence. To identify the patients' needs and provide care of good quality, the participants considered sufficient competence to be a must. Unfortunately, the lack of qualified nurses was constantly present, so that was not always the way it worked. The participants, however, claimed that it was their duty as educated nurses to transfer their own competence to less qualified colleagues: “There are several nurses in the homecare services that are not educated nurses. There are actually too few of the educated ones. But then I think that it is our responsibility to transfer our competence to those who haven't got that competence if they have to go to the patient as the first ones.” The participants described this entire situation as a kind of paradox, as working in the homecare services mainly meant to work alone and the homecare nurses should, therefore, have the best formal competence of all. Instead, they had to accept that nurses in the homecare services often had low formal competence. The participants also experienced that they were offered few possibilities for further professional development or in-service training. One of the participants described it like this: “We are left behind compared to those who work in the hospitals.”

The fact that it was challenging to maintain continuity in the care of patients in transition made the documentation and exchange of information particularly important. The participants considered the amount of time, as well as the professionals' varying competence to document and transfer the information in one way or another as essential for providing quality care. A number of the participants stressed how new computer technology constantly challenged the documentation and the transfer of information and, accordingly, could represent a threat to the care. To avoid this, they perceived that it was critical to learn and be familiar with new technology. Patients in transition were regarded as particularly vulnerable to incomplete documentation and transfer of information. Competence in this respect was a necessity to maintain continuity in the care and to establish trust and safety in the relationship with the patient. One of the participants explained it this way: “We have to be in agreement about what to do and know why we do it this or that way. To document and exchange information about this is crucial. If we all make things in our own ways we make the patient feel unsafe. It is important with continuity!”. 

### 3.5. Main Interpretation: Endeavoring to Provide Excellent Care Means Balancing between Values in Competition

The care for older persons in transition to receive homecare is characterized by ambitions to understand the patients' experiences of the transition in a larger context and to provide care that is founded in the patients' life world. To manage this, nurses endeavour to establish a trusting relationship with the patients where their qualities as human beings as well as professionals are considered significant. To provide person oriented and individualized care, they consider the patients' habits, life experiences, and autonomy as crucial. The realization of these ideals is, however, threatened by a context which is often based upon different values than those of the nurses and is characterized by insufficient resources. The nurses, thus, have to balance between competing values in the caring context while endeavoring to provide excellence care.

## 4. Discussion

The findings in this study focus on ambitions as well as on challenges that registered nurses experienced when they endeavoured to provide excellent care for older persons in transition to receive home care. Excellent care had a strong emphasis on person oriented individualized care and was based upon a relationship of trust. Consequently, their caring goals were in accordance with several recent Norwegian policy statements, including Reports no. 47 2008-2009 and no. 25 2005-2006 to the Storting [2, 3]. Results from several studies also indicate that nurses, when caring for older persons in different health care settings, perceive the same basic factors as significant in the performance of quality or excellent care [14, 17, 29].

The manner in which the participants described person oriented and individualized care in this study may to a certain extent be associated with person-centred care as outlined by McCormack [9] and McCormack and McCance [30]. According to McCormack [9], being person-centred requires the establishment of a therapeutic narrative between the professional and the patient that is built on mutual trust, understanding, and shared knowledge. The nurses who participated in this study stressed the importance of taking the patients' previous transition history into account as it constituted a vital part of the context for their understanding and for further relationship and care. The interpretation of this can be that they regarded the transition process as already begun and the patients' lives as already being disrupted and changed in one way or another. Their endeavors to obtain insight into how the previous process had affected the patients and made them vulnerable may be considered as the first cornerstone to building a therapeutic narrative. To be attentive to the recent past and see it as part of the transition process may be crucial in the care for patients in transition to receive homecare. Through their narratives, the participants contributed to a deeper insight into the concept of transition from independence to needing care, not only regarding the time perspective but also regarding the contents. Their nuanced views and descriptions indicate that they viewed the transition as complex and changing, and not as a definite stage. Therefore, their care had to be consecutively assessed related to the changing health condition, experiences, and needs of the patients. 

Chick and Meleis [31] touch on this theme when they stress the importance of understanding both the separate parts and the total phase of the transition. Further, they point out the significance of promoting greater synchrony between the time orientation of nurse and client as it will provide a basis from which to more accurately define the patients' needs. The same authors claim that it is significant to recognize or to meet the patients' needs at the point where the patients are in their perceptions of the situation. To understand where the patients are and how they perceive their situations in the initial phase of the transition to receive homecare, it might be vital to take their transition history or recent past into consideration. 

The participants in this study maintained that factors such as the transfer of patients at appropriate times, adequate information, and good communication with significant others across the health care institutions were decisive for having an appropriate start on the relationship with the patient. However, the insufficiencies they experienced in that respect were recognized as potential threats in the initial phase of transition. The cooperation in this area had, in their view, a great potential for improvement. Studies of transitional care on a system level support these concerns and discuss similar barriers for the improvement of transitional care [22–24]. These studies also state that many professionals involved in transitional care never have practiced in the settings to which they further transfer the patients. The professionals are, therefore, unfamiliar with the capacity of these settings for delivering care and are transferring patients inappropriately [23].

McCormack [9] argues that nurses have to consider the person's life as a whole in order to help to sustain meaning in life. This means clarifying beliefs and values to maximize the potential for growth and development. These ideals can be recognized in the narratives of the participants in the present study, and they were particularly aware of these ideals in the transition process. Through their attitudes and ways of caring, the nurses wanted to see and meet the patients as genuine persons with their life experiences. They strived to adapt the care to the patients' own habits and life world, and they also stressed the importance of including and supporting next of kin in a positive way. Similarly, they were conscious about the patients' rights to self-determination in relation to next of kin as well as to themselves as nurses. If they had to, the nurses negotiated with the patients to provide them the opportunity to make decisions that made the best sense to them. The way in which the participants understood “the patients' resources” can further be associated with McCormack's [9] emphasis on the importance of considering the knowledge and experience that each person brings into the care situation as central and necessary for making decisions that will best serve the patients' wellbeing. The nurses participating in this study discussed the patients' experiences and knowledge as a significant part of their resources, and they regarded these resources as a means to have an individual approach to the care. Using McCormack's [9] term, the nurses endeavoured “to develop the therapeutic narrative” between them and the patients, building upon respect for the independent individual as a living whole. Their ideals and aims were, however, challenged during the patients' transition process. 

 The transition to need professional support in the home is usually characterized by different types of changes and loss that are undesired [1]. Schumacher et al. [1] suggest that the nurse needs to address the changes and developments in the patient's situation as well as to provide an ongoing assessment. Further, they describe the following processes that nurses must provide to proceed in the right direction, namely, to help the patients to redefine meaning, to modify expectations, to develop new skills and knowledge, to maintain continuity, and to find opportunities for personal growth. The participants in the present study described several conditions that they considered as obstacles for succeeding in such processes. Together with time and competence, continuity appeared to be the most important factor. Due to the organizational arrangements and the large number of health care providers, it was almost impossible to maintain continuity. In the light of McCormack's descriptions of person-centred care [9] and also in the light of the transition theory [31], continuity in the care of persons in transition to receive home care must be considered as one of the keystones for providing a healthy transition process. Therefore, the frequent changes and rotation in care personnel, as the participants experienced it, may be especially unfortunate for patients in transition. According to Chick and Meleis [31] concurrently occurring transitions compound upon one another. Changes and rotation of staff may represent unstable environments and influence how smoothly the transition is experienced. 

In their study of older persons who had received homecare between two and four weeks, Hvalvik and Reierson [11] found that the patients accepted their situation in a positive way and had strong expectations to receive care that improved their health and also involved them in an active way in the caring process. The authors suggest that this attitude may be characteristic for this group of patients in transition and hence represent a significant potential for which health care providers to be aware. To take advantage of this attitude, nurses must be given the opportunity to establish the therapeutic narrative that McCormack [9] describes. In addition, in this respect, sufficient time, continuity, and competence are urgent elements.

As we already have stated, one important goal brought out in the Norwegian Coordination Reform [2] is that older patients shall be quickly discharged and returned to his or her residential municipality after completing treatment in the specialist care. A smooth transition process may prevent complications and rehospitalization with unfortunate consequences first and foremost for the patient, and also for society. Coleman and Berenson [23] state that a major barrier for improving the quality of transitional care is that it remains as an underrecognized, although significant, issue that has received almost no attention in the health policy arena. In that respect, it is of decisive value to be informed by those who are closest to the patients and obtain insight into their important experiences from the actual transition process.

## 5. Conclusion

The results from this study indicate that the nurses endeavoured to provide person-oriented and individualized care for older persons in transition to receive homecare. The type of care they aimed to provide was in accordance with national and international health related goals, as well as with theories of healthy transitions. Their efforts and ambitions to provide excellent care were, however, challenged and threatened by various circumstances in the caring context. The cooperation across organizational levels was pointed out as a critical factor with potential for improvement. In addition, the need for greater consciousness regarding time, competence, and continuity in the care was emphasized. The results illustrate that nurses are flexible and have to balance competing values in the health care system. To facilitate for a context that supports the nurses' ambitions of excellent care is decisive for the patients who are in transition to receive homecare, and also for those who are responsible for the performance of care, as it will represent an advantage to the health care service and society as well.

As we see it, this study has contributed to a more complex view on the concept of transition from being independent to needing care among older people. The concept, however, requires further exploration in future research. To explore and describe the complexity and variations in this stage of life would be of particular interest for the field of nursing practice, and for theory development. In addition, further research focusing on problematic transitions from self-supported to supported living would be a significant contribution to reach the goals of nursing therapeutics from a transition perspective.

## Figures and Tables

**Table 1:** An illustration of the themes, variations, and interpretation emerged from the analysis.

Themes	Variations	Interpretations
The beginning of the transition process	Time perspective Marker events Experiences Involvement and role State of health Attitudes to receive help	It is essential to have insight into the patients' transition history

To interpret and act in correspondence with the individual patient's situation	Personality Consciousness Communication Courage Professionalism Responsibility Empathy Attention Acting independently	The nurse' repertoire is challenged in the transition process

Considering the patient and the person as recipient of home care	Habits Exchanges Everyday life Wholeness Motivation Resources Autonomy Dependency Next of kin	Care must be adapted to the patients' life world

To provide person oriented and individualized care is dependent upon internal and external conditions	Cooperation Competence Continuity and time Documentation and information	The excellence of care is challenged by the context
